# Epidemiology of sepsis in Catalonia: analysis of incidence and outcomes in a European setting

**DOI:** 10.1186/s13613-017-0241-1

**Published:** 2017-02-20

**Authors:** Juan Carlos Yébenes, Juan Carlos Ruiz-Rodriguez, Ricard Ferrer, Montserrat Clèries, Anna Bosch, Carol Lorencio, Alejandro Rodriguez, Xavier Nuvials, Ignacio Martin-Loeches, Antoni Artigas, SOCMIC (Catalonian Critical Care Society) Sepsis Working Group, Abdo Taché, Antoni Margarit, Assumpta Ricart, Adolf Ruiz-Sanmartin, Begoña Balsera, Berta Cisteró, Candelària de Haro, Concepció Rovira, Eva Torrents, Francisco Álvarez-Lerma, Herbert Baquerizo, Joan Balcells, José L. Echarte, José Luna, Josep M. Sirvent, Juan Méndez, Lluís Zapata, Lluïsa Bordejé, Lourdes Jiménez, Maite Martínez-Izquierdo, María L. Martínez, María P. Gracia-Arnillas, Mercedes Palomar, Miguel Sánchez, Pablo Pujol, Pau Garro, Pau Torrabadella, Paula Vera, Roger Bisbal, Ruth Hernández, Teresa M. Tomasa, Víctor Pérez-Claveria

**Affiliations:** 1 Critical Care Department, Hospital de Mataró, Mataró, Spain; 2 Grup de Recerca en Sepsis, Imflamació i Seguretat (AGAUR 2014-SGR926), Barcelona, Spain; 3 Escola Superior de Ciencies de la Salut, Universitat Pompeu Fabra, Mataró, Spain; 4 Critical Care Department, Vall d’Hebron University Hospital, Barcelona, Spain; 5 Shock, Organ Dysfunction and Resuscitation (SODIR) Research Group, Vall d’ Hebron Research Institut, Universitat Autònoma de Barcelona, Barcelona, Spain; 6 CIBER Enfermedades Respiratorias, Madrid, Spain; 7 Divisió d’Anàlisi de la Demanda i l’Activitat, Servei Català de la Salut (CatSalut), Barcelona, Spain; 8 Critical Care Department, Hospital Universitari Dr. Josep Trueta, Girona, Spain; 9 Critical Care Department, Hospital Universitari Joan XXIII, Tarragona, Spain; 10 IISPV/URV, Tarragona, Spain; 11 Critical Care Department, Hospital Universitari Arnau de Vilanova, Lleida, Spain; 12 Institut de Recerca Biomèdica (IRB), Lleida, Spain; 13 Multidisciplinary Intensive Care Research Organization (MICRO), St James’s University Hospital, Trinity Centre for Health Sciences, Dublin, Ireland; 14 Critical Care Center, Sabadell Hospital, Corporació Sanitaria Universitaria Parc Tauli, Sabadell, Spain; 15 Universitat Autonoma de Barcelona, Sabadell, Spain; 16 Servei de Medicina Intensiva, Hospital de Mataró, Carretera de Cirera s/n, 08304 Mataró, Spain

**Keywords:** Sepsis, Septic shock, Mortality, Epidemiology

## Abstract

**Background:**

Up-to-date identification of local trends in sepsis incidence and outcomes is of considerable public health importance. The aim of our study was to estimate annual incidence rates and in-hospital mortality trends for hospitalized patients with sepsis in a European setting, while avoiding selection bias in relation to different complexity hospitals.

**Methods:**

A large retrospective analysis of a 5-year period (2008–2012) was conducted of hospital discharge records obtained from the Catalan Health System (CatSalut) Minimum Basic Data Set for Acute-Care Hospitals (a mandatory population-based register of admissions to all public and private acute-care hospitals in Catalonia). Patients hospitalized with sepsis were detected on the basis of ICD-9-CM codes used to identify acute organ dysfunction and infectious processes.

**Results:**

Of 4,761,726 discharges from all acute-care hospitals in Catalonia, 82,300 cases (1.72%) had sepsis diagnoses. Annual incidence was 212.7 per 100,000 inhabitants/year, rising from 167.2 in 2008 to 261.8 in 2012. Length of hospital stay fell from 18.4 to 15.3 days (*p* < .00001), representing a relative reduction of 17%. Hospital mortality fell from 23.7 to 19.7% (*p* < .0001), representing a relative reduction of 16.9%. These differences were confirmed in the multivariate analysis (adjusted for age group, sex, comorbidities, ICU admission, emergency admission, organ dysfunction, number of organ failures, sepsis source and bacteraemia).

**Conclusions:**

Sepsis incidence has risen in recent years, whereas mortality has fallen. Our findings confirm reports for other parts of the world, in the context of scarce administrative data on sepsis in Europe.

**Electronic supplementary material:**

The online version of this article (doi:10.1186/s13613-017-0241-1) contains supplementary material, which is available to authorized users.

## Background

Sepsis has been recently redefined as an infection that leads to organ dysfunction [1]. Although a hospitalized patient with sepsis is more likely to die than a patient with heart attack or stroke [2], sepsis is still not evaluated with the same urgency as other critical conditions. Sepsis mortality can be reduced considerably by adopting early recognition protocols and using standardized emergency treatment, but fewer than 1 in 7 patients are actively intervened in this way [3]. Treatment ineffectiveness is often due to late sepsis diagnosis—mainly a failure by caregivers or healthcare professionals to suspect sepsis. The clinical symptoms and laboratory signs currently used for diagnostic purposes are not specific for sepsis, and there is a lack of reliable systems for timely detection of septic patients.

Proper detection of sepsis and its progression are essential to patient management. Epidemiology case studies using administrative hospital data have reported both growing incidence and declining mortality rates associated with severe sepsis in several different countries but mainly in the USA, Australia and New Zealand [4–11]. However, administrative data may be affected by changes in coding practices that distort incidence and mortality estimates. Recently, Stevenson et al. [12] compared incidence and mortality trends in trial data with those observed in administrative data, observing that, since 1991, risk-standardized 28-day severe sepsis mortality has tended to decline in parallel for both methods; this which would indicate that trends in severe sepsis mortality as calculated from administrative data and International Classification of Diseases, 9th revision, Clinical Modification (ICD-9-CM) algorithms are likely to be accurate.

Catalonia, an autonomous region in Spain, has actively begun to advocate more vigorous efforts to decrease the sepsis burden through the development of an Inter-hospital Sepsis Emergency Code, operational between the Catalan Health Service (CatSalut) and seven local medical societies [13]. Since the identification of trends in sepsis outcomes is of considerable public health importance, the aim of our study was to estimate population and annual in-hospital incidence rates and in-hospital mortality trends for patients with sepsis between 2008 and 2012 in Catalonia, before implementation of the Inter-hospital Sepsis Emergency Code designed to coordinate and optimize care of patients with sepsis.

## Methods

### Data sources

A retrospective analysis was conducted of hospital discharge records from the Minimum Basic Data Set for the Catalan Health System (CatSalut) Acute-Care Hospitals (a mandatory population-based register of admissions to all public and private acute-care hospitals in Catalonia) enables evaluation and optimization of resource use, provides support to and improves healthcare planning and facilitates procurement management and payments. To ensure data quality, the CMBD-HA input data are systematically validated internally and periodically validated externally. The data set contains demographic and clinical data for patient care episodes, including age, sex, length of stay (days), one primary diagnosis, up to nine secondary diagnoses, one primary procedure, up to seven secondary procedures and status on discharge (alive or dead). Official data from the register of insured persons maintained by CatSalut were used to estimate crude and specific hospitalization rates (universal coverage for 7,601,813 inhabitants in 2012).

### Patients

Sepsis, formerly severe sepsis [1] was defined by the presence of infection and at least one organ dysfunction. Patients hospitalized with sepsis were detected, according ICD-9-CM codes used to identify acute organ dysfunction and infectious processes following the Angus methodology [5], over a 5-year period (2008–2012). To avoid overlaps, we excluded patients who were transferred from one acute-care hospital to another during the same severe sepsis episode.

### Coding

Diagnoses and procedures were coded using the ICD-9-CM, whose codes to identify patients with sepsis were updated in 2000 to the following: 995.91 (sepsis), 995.92 (severe sepsis) and 785.52 (septic shock) (Supplementary Appendix: Additional file 1). Although information was not available regarding the unit or department where patients were treated (intensive care unit (ICU), internal medicine unit, etc.), we indirectly deduced ICU admission from procedures typically used in intensive care management (Supplementary Appendix: Additional file 1). The Charlson comorbidity index with its 17 comorbid disease categories [13] was used to assess the presence of underlying comorbidities. The ICD-9-CM codes used to identify acute organ dysfunction and infectious processes are listed in Supplementary Appendix: Additional file 1.

### Statistical analysis

The hospitalization rate was defined as the yearly number of admissions per 100,000 population (excluded were 1590 admissions from non-residents in Catalonia). Crude overall and specific hospitalization rates by age and sex were calculated. Continuous variables and discrete variables were compared using analysis of variance and the Chi-square test, respectively. Multivariate logistic regression, adjusted for other significant variables, was used to analyse hospital mortality risk by year of admission for the study population and for the ICU and non-ICU patient groups; variables were entered one by one and retained when their significance was <.10 and were clinically plausible. For the regression analysis, each of the clinical attributes included (comorbidities, acute organ failure and infection) were treated as binary (dummy) variables indicating the presence or absence of these conditions; a single patient could therefore account for more than one attribute. The area under the receiver operating characteristic curve (AUROC) was used to evaluate how well the multivariate logistic regression model discriminated between patients with severe sepsis who were discharged alive versus those who died in-hospital [14]. Data analysis was performed using SPSS 18.0 software (SPSS Inc, Chicago, IL, USA).

## Results

### Incidence and main features of severe sepsis

Of 4,761,726 discharges from all acute-care hospitals during the study period, 82,300 (1.72%) had sepsis. Demographic characteristics and comorbidities for patients with sepsis are shown in Table 1.

**Table 1:** Profile of patients with severe sepsis in Catalonia 2008–2012

	Total *N* = 82,300	Alive on discharge *N* = 64,511	Dead on discharge *N* = 17,789	*p*
	Mean (SD)	Mean (SD)	Mean (SD)	
Age (years)	71.2 (19.7)	70.6 (20.4)	73.3 (16.6)	<.0001
Length of hospital stay (days)	16.7 (19.5)	16.8 (19.3)	16.3 (20.3)	<.001
Charlson comorbidity index	5.1 (2.6)	5.0 (2.6)	5.6 (2.7)	<.0001

	%	%	%	*p*
Age (years)				
<15	3.2	3.7	1.4	<.0001
15–44	6.1	6.6	4.2	
45–64	16.4	16.2	17.3	
65–74	17.5	17.3	18.4	
75–84	32.9	32.8	33.6	
>84	23.8	23.5	25.0	
Sex				
Males	56.7	56.1	59.0	<.0001
Comorbidities				
Chronic kidney disease	23.8	24.5	20.9	<.0001
COPD	22.4	22.9	20.6	<.0001
Cancer	14.5	12.5	21.5	<.0001
Metastasis	4.8	5.8	8.3	<.0001
Peripheral vascular disease	4.4	4.2	4.9	<.0001
Complicated diabetes	4.0	4.3	3.0	<.0001
Liver disease: mild	9.7	8.7	13.1	<.0001
Liver disease: moderate–severe	3.0	2.4	4.9	<.0001
Myocardial infarction	3.8	3.6	4.7	<.0001
Congestive heart failure	20.1	19.1	23.8	<.0001
Cerebrovascular disease	6.7	6.5	7.6	<.0001
AIDS/HIV infection	1.0	0.9	1.3	<.0001
Emergency stay (%)	89.4	89.8	87.8	<.0001
ICU admissions	28.2	22.3	49.8	<.0001
Admission year				
2008	15.6	15.1	17.1	<.0001
2009	17.9	17.4	19.6	
2010	19.7	19.6	20.0	
2011	22.3	22.6	21.0	
2012	24.6	25.2	22.3	
Sepsis origins				
Urinary	37.2	41.1	23.0	<.0001
Respiratory	32.5	31.4	36.4	<.0001
Abdominal	11.0	10.1	14.3	<.0001
Skin and soft tissues	4.1	4.2	3.9	.06
Endocarditis	1.6	1.5	2.0	<.0001
Device-related	1.1	1.2	0.6	<.0001
CNS	0.9	0.9	1.2	<.0001
Others	37.9	41.0	26.5	<.0001
Bacteraemia	24.7	19.1	44.9	<.0001
Organ dysfunction				
Kidney	58.4	56.9	63.7	<.0001
Lung	20.5	15.7	37.9	<.0001
CNS	19.7	21.6	12.7	<.0001
Haematologic	11.1	11.0	11.5	.054
Cardiovascular	4.9	5.2	3.6	<.0001
Liver	1.3	0.7	3.3	<.0001
Number of organ failures				
1	86.3	89.7	72.9	<.0001
2	12.0	9.4	21.8	
3	1.6	0.8	4.7	
4 or more	0.2	0.1	0.6	

Data are presented as mean and standard deviation or %

*AIDS* acquired immune deficiency syndrome, *CNS* central nervous system, *COPD* chronic obstructive pulmonary disease, *HIV* human immunodeficiency virus, *ICU* intensive care unit

**Fig. 1:**
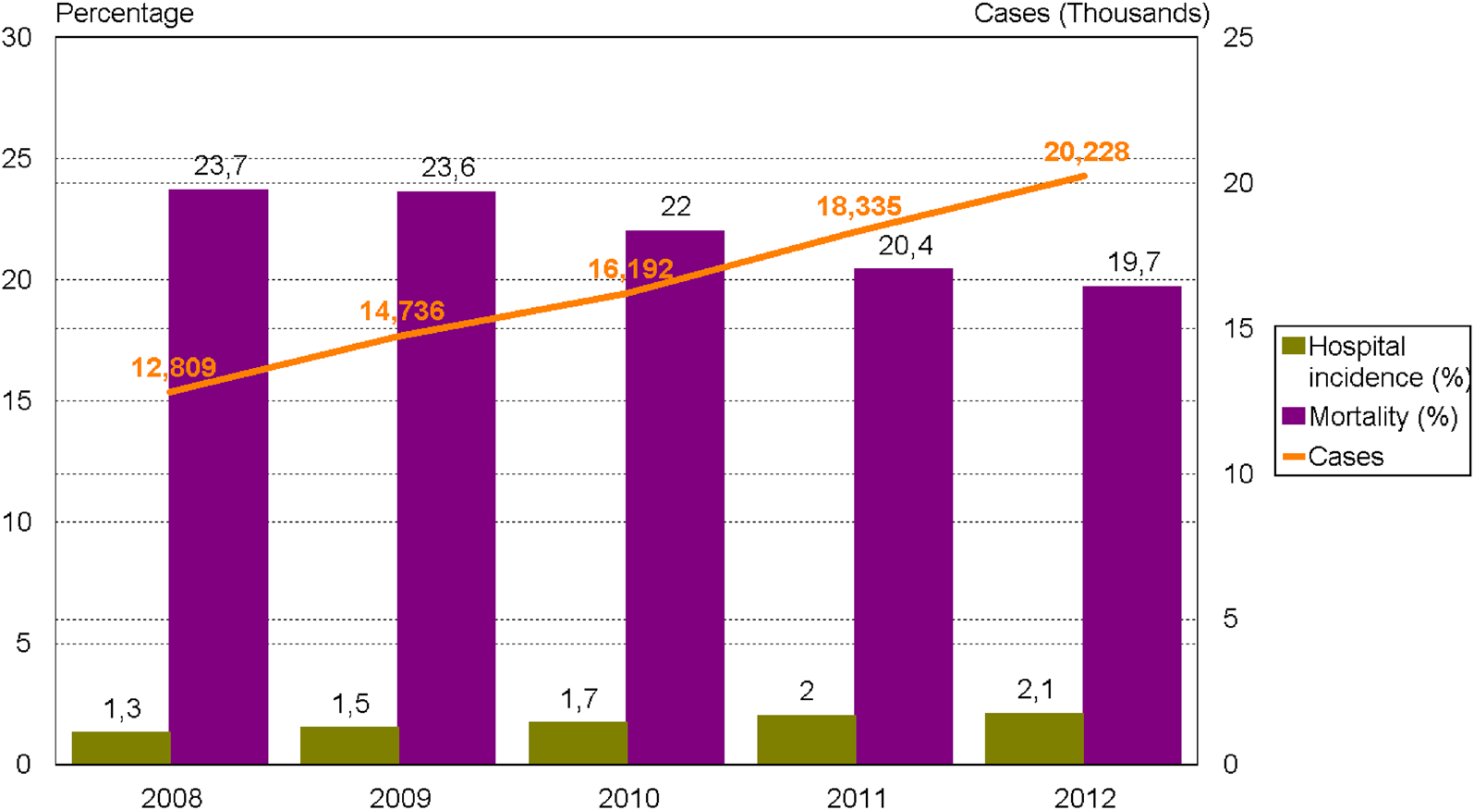
Number of cases, mortality rates and hospital incidence rates for sepsis in Catalonia (2008–2012). Incidence of sepsis increased from 12,809 cases to 20,228 cases in the 5-year study period (mean 16,460 cases per year), representing 1.3 and 2.1% (*p* < .0001) of hospital admissions and an average yearly increase of 6%. However, hospital mortality decreased from 23.7 to 19.7% (*p* < .0001) for a yearly relative reduction of 3.4%

**Fig. 2:**
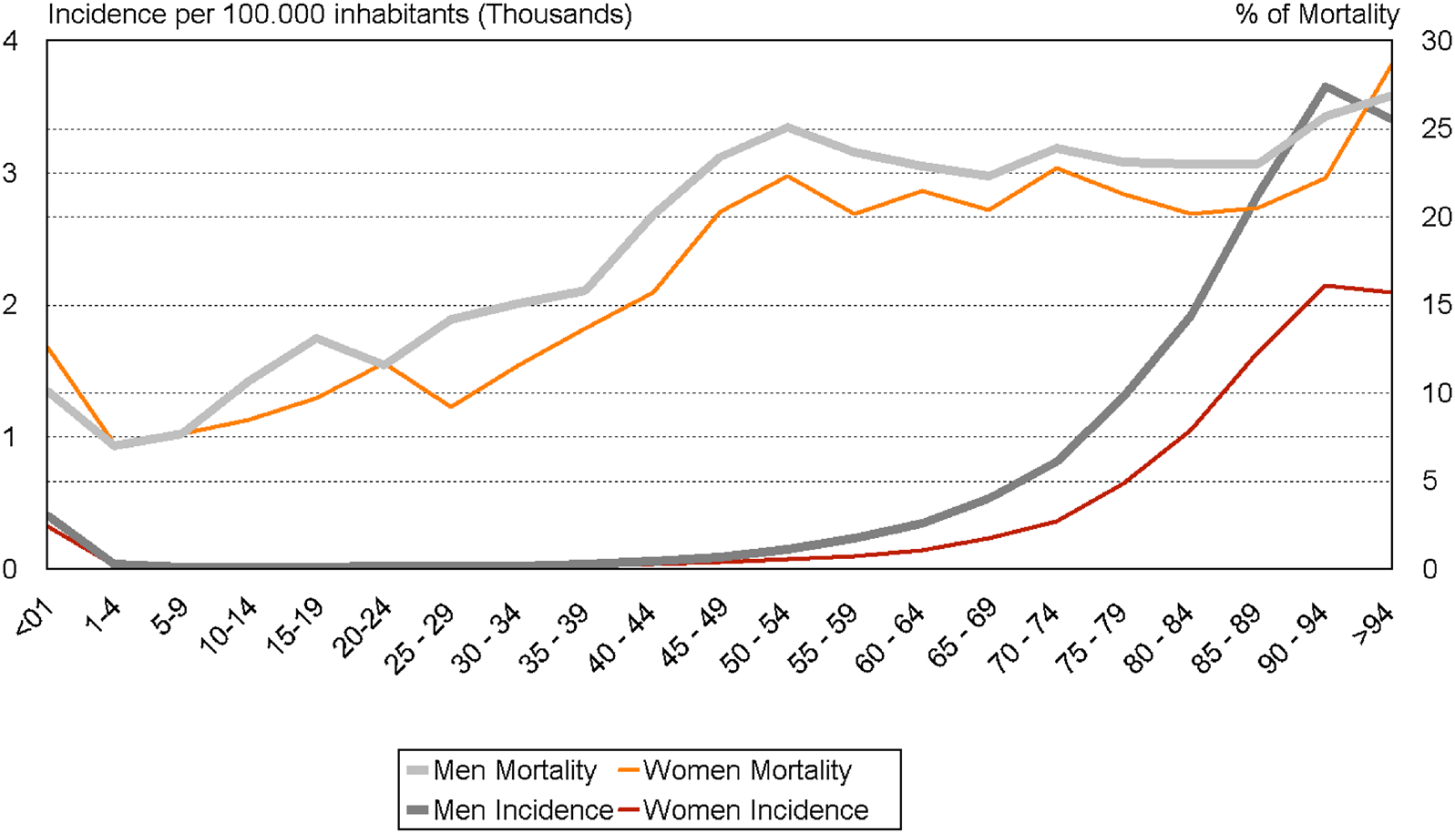
Age-specific incidence and mortality rates for all cases of severe sepsis by sex in Catalonia (2008–2012). The *dark line* represents incidence (*thicker* for men and *thinner* for women) expressed as a thousand cases per 100,000 inhabitants. Age-adjusted mortality is expressed as the number of deaths with respect to the number of cases grouped according to 5-year age brackets

Annual incidence in the population was 212.7 per 100,000 inhabitants/year, increasing from 167.2 in 2008 to 261.8 per 100,000 inhabitants/year in 2012 (Fig. 1). Sepsis was significantly associated with age (more frequent in older patients) and sex (208.3 cases for men versus 156.0 cases for women per 100,000 inhabitants/year) (Fig. 2).

The most frequent origins of sepsis were urinary and respiratory tract infections, accounting for 37.2 and 32.5% of cases, respectively, followed by the abdomen (11%). Almost a quarter (24.7%) of cases presented bacteraemia. Acute kidney injury was the most frequent organ failure (58.4%), followed by respiratory failure (20.5%) and central nervous system failure (19.7%). Cardiovascular dysfunction was reported in 4.9% of cases. Two or more acute organ failures were documented in 14% of cases (Table 1).

### Hospital outcomes

In-hospital mortality over the study period was 21.6% (95% confidence interval (CI); 18.6–24.9). Hospital mortality was higher in older patients with higher Charlson comorbidity score, in patients with bacteraemia (39.3% in patients with positive versus 15.8% in patients with negative blood cultures), and was also higher in patients with more organ failures (three or more, 63.4%). Respiratory and abdominal origins were associated with higher mortality (24 and 28%, respectively). Mean (SD) length of stay was 16.7 (19.5) days, with no clinically relevant differences for patients who died in-hospital versus who were discharged alive (despite a value of *p* < .001) (Table 1 and Table 2).

**Table 2:** Overall mortality by patient characteristics, sepsis origins, presence of bacteraemia and organ dysfunction by year of admission in Catalonia (2008–2012)

Condition	*N*	Presence of condition (%)						In-hospital mortality (%)
		2008	2009	2010	2011	2012	*p*	
Mortality		23.7	23.6	22.0	20.4	19.7	<.0001	
Source of sepsis								
Urinary tract	30,600	33.6	35.0	37.1	39.8	38.8	<.0001	13.9
Respiratory tract	26,748	34.2	34.0	31.2	30.9	32.7	<.0001	24.2
Abdomen	9065	10.7	11.2	11.3	11.0	10.9	NS	28.0
Skin and soft tissues	3394	3.9	4.0	4.0	4.4	4.2	NS	20.3
Endocarditis	1326	1.8	1.8	1.9	1.4	1.4	<.0001	27.3
Device-related	869	1.1	1.1	1.2	1.1	0.9	.016	11.6
CNS	775	1.2	1.1	1.1	0.7	0.7	<.0001	27.5
Other	31,149	37.4	37.5	39.2	38.1	37.1	.001	15.1
Bacteraemia	20,285	25.5	25.5	24.5	24.8	23.5	<.0001	39.3
Organ dysfunction								
Kidney	48,072	50.8	53.5	57.1	61.9	64.7	<.0001	23.6
Respiratory	16,876	26.1	24.1	20.9	17.9	16.3	<.0001	40.0
CNS	16,177	20.5	19.6	19.1	18.9	20.3	<.0001	13.9
Haematologic	9158	11.6	12.3	12.2	11.2	9.1	<.0001	22.4
Cardiovascular	3988	5.3	5.4	4.7	4.6	4.4	<.0001	16.1
Liver	1058	1.0	1.3	1.3	1.3	1.4	.029	55.9
Number of organ failures								
1	70,874	86.6	85.9	86.5	86.0	85.8	NS	18.5
2	9964	11.7	12.2	11.8	12.4	12.3		39.0
3 or more	1462	1.7	1.9	1.9	1.7	1.8		63.4

Data are presented as number of cases or %

*CNS* central nervous system, *NS* non-significant

### Incidence and in-hospital outcome trends

**Fig. 3:**
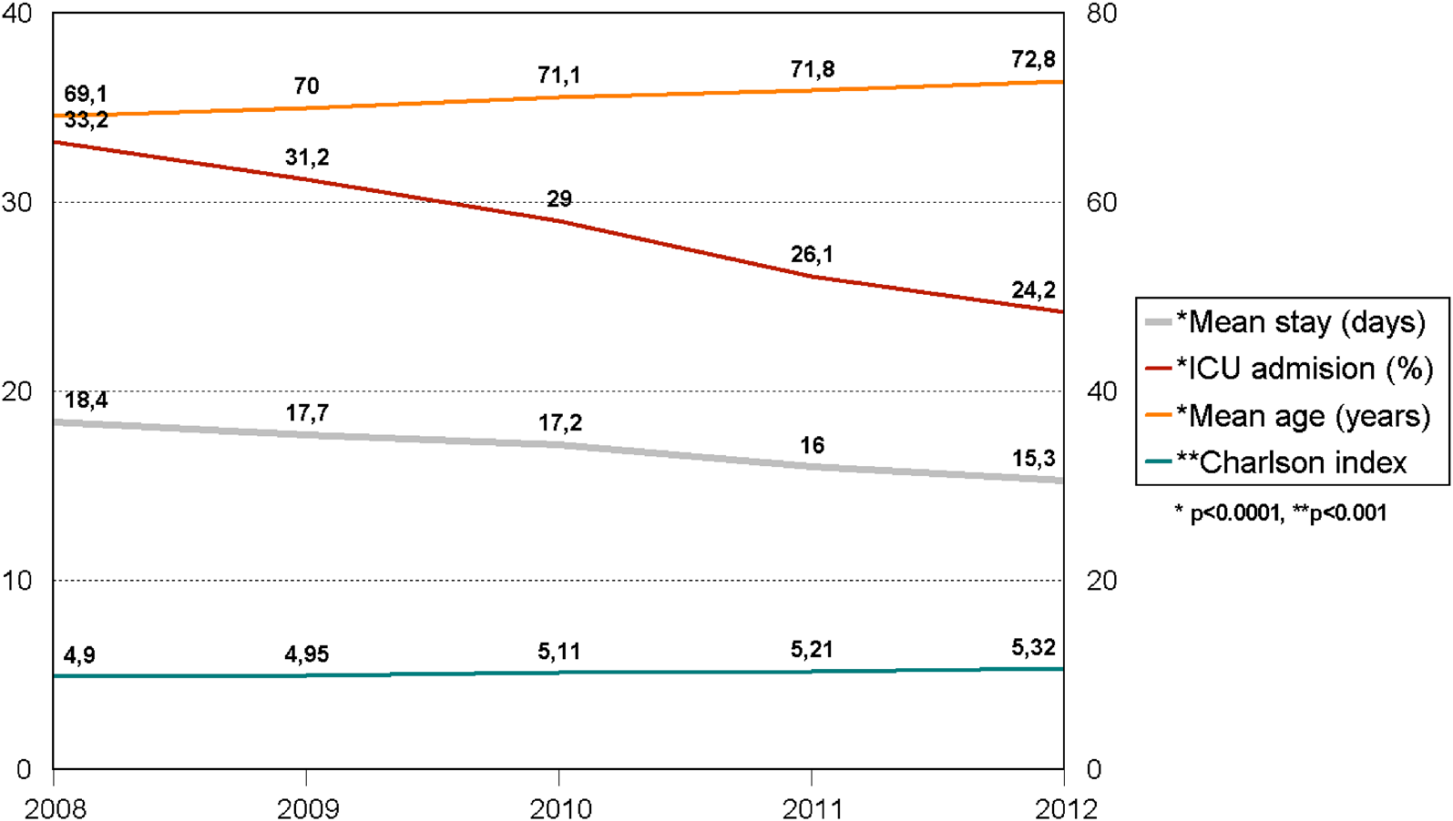
Trends for main characteristics and hospital stay for patients with sepsis in Catalonia (2008–2012)

Incidence of sepsis increased in the five-year study period from 12,809 cases to 20,228 cases (mean 16,460 cases per year over the period), representing 1.3 and 2.1% (*p* < .0001) of hospital admissions, respectively (Fig. 2). Observed in the same period were an increase in mean age, from 69.1 to 72.8 years (*p* < .0001), and an increase in mean Charlson comorbidities score, from 4.9 to 5.3 (*p* < .001) (Fig. 3).

Length of hospital stay decreased from 18.4 to 15.3 days (*p* < .0001), representing a relative reduction of 17% (Fig. 3). Univariate analysis showed that hospital mortality also decreased—from 23.7 to 19.7% (*p* < .0001)—for a relative reduction of 18.6% (Fig. 2). These differences were confirmed in the multivariate analysis adjusted for all significant variables (age group, sex, comorbidities, ICU admission, emergency admission, organ dysfunction, number of organ failures, infection source and presence of bacteraemia) (Tables 1, 2). Differences between 2008 and all the ensuing years except 2009 were statistically significant. The logistic regression (reference year 2008) indicated a mortality odds ratio (OR) for patients with sepsis in 2012 of 0.772 (95% CI 0.727–0.820) (Table 3). The falling trend in the mortality OR was linear throughout the study period for all patients with sepsis, whether or not treated in the ICU. Values for the AUROC were calculated to evaluate how well the multivariate logistic regression model discriminated between patients discharged alive and discharged dead: 0.782 (95% CI 0.779–0.786) for all patients, 0.746 (0.741–0.752) for non-ICU patients and 0.749 (0.743–0.756) for ICU patients.

**Table 3:** Univariate and multivariate analyses of in-hospital mortality by year of admission in Catalonia (2008–2012)

	Univariate analysis			Multivariate analysis		
	*N*	% mortality	*p*	OR	95% CI for OR	*p*
All patients *N* = 82,300						
2008	12,809	23.7	<.0001	1	–	–
2009	14,736	23.6		0.988	0.928–1.052	NS
2010	16,192	22.0		0.911	0.856–0.969	.003
2011	18,335	20.4		0.818	0.770–0.870	<.0001
2012	20,228	19.7		0.772	0.727–0.820	<.0001
Non-ICU patients *N* = 59,064						
2008	8551	15.8	<.0001	1	–	–
2009	10,137	16.3		1.028	0.945–1.117	NS
2010	11,497	15.4		0.941	0.867–1.022	NS
2011	13,545	14.8		0.859	0.793–0.931	<.0001
2012	15,334	13.9		0.769	0.711–0.832	<.0001
ICU patients *N* = 23,236						
2008	4258	39.5	.002	1	–	–
2009	4599	39.6		0.938	0.854–1.031	NS
2010	4695	38.2		0.868	0.790–0.953	.003
2011	4790	36.0		0.757	0.686–0.832	<.0001
2012	4894	37.6		0.778	0.702–0.855	<.0001

Data are presented as number of death or %. The multivariate analysis is adjusted by sex, age group, comorbidities, ICU admission, emergency admission, organ dysfunction, number of organ failures, septic origins and bacteraemia

*CI* confidence interval, *ICU* intensive care unit, *OR* odds ratio, *NS* non-significant

## Discussion

Most epidemiological data on sepsis refers to the first decade of twenty-first century and almost exclusively refer to the USA. This is a large observational study of patients discharged from all national health system acute-care hospitals conducted in a European setting. We estimated the mean sepsis incidence to be 212.7 cases per 100,000 inhabitants/year and in-hospital mortality to be 21.6%. Incidence and mortality varied over time, with a yearly increase in incidence of 7.3%, a yearly relative reduction of 3.3% in length of stay and a yearly reduction in in-hospital mortality of 3.4%. After adjustments for relevant clinical and epidemiological variables, the reduction in mortality remained statistically significant.

The estimated incidence of sepsis in our study was lower than reported in the USA and slightly higher than reported in smaller European studies [2, 6, 7]. Previous studies conducted in Spain reported incidences of between 110 and 230 cases per 100,000 inhabitants/year [15, 16], versus the 212.7 cases observed in our study. Differences in calculated incidences may be related to structural or functional organization [17] or may be due to discharge diagnosis coding. Nonetheless, we would like to emphasize the importance of using local data to monitor trends in activity and results over time. Moreover, the number of sepsis cases in our study increased yearly, a finding which is consistent with findings reported in other epidemiological studies [10–12].

Estimates of sepsis incidence and trends are also essential to estimate the resources needed to care for these patients. Sepsis incidence is increasing compared to incidence for other leading causes of mortality such as acute myocardial infarction or ischaemic stroke. The CatSalut data on hospital admissions/year for severe sepsis (five-year mean, 16,460 cases) are close to the combined numbers for acute coronary syndrome and ischaemic stroke admissions together, at 11,000 and 8000, respectively [18, 19]. However, incidence rates for acute coronary syndrome and ischaemic stroke, unlike for sepsis, are stable [19–21]. In the USA, the percentage of septic patients with a fatal outcome increased from 14% in 2000 to 16% in 2010; in contrast, mortality for respiratory failure decreased from 25 to 17%, for heart attack from 10 to 8%, for cancer from 8 to 4% and for stroke from 6 to 5% [22].

Prospective versus retrospective analysis observed differences in incidence and source of sepsis. Prospective monitoring is laborious, costly and complex and can also be affected by issues such as inclusion criteria or data sources [23, 24]. Although retrospective analyses from hospital discharges—as in our study—can also be affected by definitions, codes and analytical methods, they serve an important function in analysing local trends and outcomes. Gaieski [24] observed a 3.5-fold difference in estimates of absolute incidence using different database abstraction methods. Nonetheless, trends were similar irrespective of the methodology. Stevenson et al. [12] recently found that severe sepsis mortality was 10% higher for patients included in the control group of clinical trials compared to administrative data (collected according to Angus’ criteria) [5]; nonetheless, mortality trends were similar, irrespective of the data source—and were also similar to the 3% yearly reduction found in our study. Stevenson et al. consequently concluded that administrative data are useful in monitoring mortality trends in patients with severe sepsis.

Length of stay and mortality both decrease yearly during the study period—to a statistically significant degree according to the multivariate analysis adjusted for demographic data, comorbidities, infection source and number of organ failures. The external validity of our findings is supported by the fact that mortality in 2008 was the same as that reports by the PROWESS-SHOCK study placebo group [25]. Other recent large randomized clinical trials (RCTs) have reported mortality rates of 18–30% [26, 27], further confirming the likely validity of 2008 as our baseline year. Kaukonen et al. [11], who recently reported similar results for Australia and New Zealand, observed an annual absolute decrease of 1.3% in risk, from 24% in 2008 to 19% in 2012. In our study, multivariate analysis revealed a robust association between mortality and year of detection, as adjusted for confounding factors including sex, age group, comorbidities, ICU admission, emergency admission, organ dysfunction, number of organ failures, septic focus and the presence of bacteraemia. Although our study was not designed to address this issue, a potential improvement in the management of sepsis could be suggested, explained in part as a consequence of training and increased clinical awareness [3, 28–30].

Our results suggest also that sepsis outcomes should be interpreted according to the year of data collection and the presence of comorbidities. Moreover, underpowered RCTs would be avoided if these effects were taken into account in estimating statistical power and sample size. Yearly reduction in crude mortality rates should be expected, bearing in mind that overestimated mortality rates may lead to underpowered studies which might, in turn, lead to potentially useful treatments being downgraded due to lack of evidence. Furthermore, excluding elderly patients and patients with comorbidities from RCTs represents a form of selection bias; Kaukonen et al. [11], for instance, reported a 4.6% mortality rate for comorbidity-free patients and young patients (versus our rate of 21.6%). Another issue is that sepsis outcomes are too often viewed as binary: The patient dies (failure) or survives (success). Studies also tend to focus on in-hospital mortality and length of stay as an outcome measure for ICU patients, overlooking the fact that many patients admitted for sepsis die after discharge. There is an unmet need to improve knowledge regarding long-term effects in patients with sepsis [1–8], so other outcome indicators such as long-term morbidity and quality of life are likely to be included in future trials.

Just under a quarter (24.7%) of our patients presented with bacteraemia, associated with higher mortality. Patients with bacteraemia could represent a suitable population to monitor prospectively in clinical practice, as bacteraemia, unlike sepsis, is easily identified retrospectively, is easily distinguished from other non-infectious diseases that cause organ dysfunction and is also easily stratified using the sepsis-related organ failure assessment (SOFA) instrument [31].

The main strengths of our study are the large cohort of patients included in a European setting, the fact of including 100% of admissions to both public and private hospitals of Catalonia Health System, the long period of data collection and the use of a previously validated strategy. Our study has several limitations. The fact that cases of sepsis were identified indirectly using ICD-9-CM codes implies less accuracy in identifying cases and a poor clinical analysis compared to prospective methods. Urinary infections appear as the main focus of sepsis in our study. The relevance of each focus can be affected by population characteristics or methodology. It also can affect incidence or severity results. Case recruitment may also have been affected by coding, as reflected in our results for cardiovascular dysfunction. Hypotension was poorly documented on discharge. Incidence, as reported in our study, probably does not reflect clinical incidence. Cardiovascular dysfunction incidence rates of 7.2–42% have been reported for epidemiological or retrospective studies, in contrast to rates of up 90% for prospective studies [4, 5, 9, 13, 15, 32, 33]. Our study cannot account for reasons for reduced mortality and shorter stays. Inclusion of a specific severity scores, such as SOFA, in the multivariate analysis would allow insights into whether mortality reduction is related to the inclusion of less severe patients. Unfortunately, our study design does not admit this conclusion. Given that the CMBD-HA does not specifically collect data about ICU admission, the category ‘ICU stay’ was deduced from procedures typically used in ICUs. We think that since this definition is highly specific but not sensitive, we cannot rule out the possibility that some septic patients with less severity were excluded from ICU admission.

## Conclusions

Sepsis incidence has risen continuously in recent years in Catalonia. While mortality and length of stay have fallen, despite increases in age and in comorbidities, our findings corroborate results reported for other parts of the world, in the context of scarce administrative data on sepsis in Europe.

## Additional file

**Additional file 1.** ICD-9-MC codes used to identify infectious process, site of infection, acute organ dysfunction and procedures used in intensive care unit.

## Abbreviations

AIDS: acquired immune deficiency syndrome
CI: confidence interval
CMBD-HA: Minimum Basic Data Set—Acute-Care Hospitals
COPD: chronic obstructive pulmonary disease
ICD-9-CM: International Classification of Diseases, 9th revision, Clinical Modification
ICU: intensive care unit
NS: non-significant
OR: odds ratio
RCT: randomized clinical trials
SD: standard deviation
SOFA: sepsis-related organ failure assessment
