# Supplementary material for: Epidemiology of sepsis in Catalonia: analysis of incidence and outcomes in a European setting
Source: Ann Intensive Care. 2017 Feb 20;7:19. doi: 10.1186/s13613-017-0241-1 (PMC5318305; doi:10.1186/s13613-017-0241-1)
Supplement: Supplementary file 1 — Additional file 1. ICD-9-MC codes used to identify infectious process, site of infection, acute organ dysfunction and procedures used in intensive care unit. [file 13613_2017_241_MOESM1_ESM.docx]

**Supplemental Appendix**

**ICD-9-MC Codes used to identify an infectious process**

001, Cholera; 002, Typhoid/paratyphoid fever; 003, Other salmonella infection; 004, Shigellosis; 005, Other food poisoning; 008, Intestinal infection not otherwise classified; 009, Ill-defined intestinal infection; 010, Primary tuberculosis infection; 011, Pulmonary tuberculosis; 012, Other respiratory tuberculosis; 013, Central nervous system tuberculosis; 014, Intestinal tuberculosis; 015, Tuberculosis of bone and joint; 016, Genitourinary tuberculosis; 017, Tuberculosis not otherwise classified; 018, Miliary tuberculosis; 020, Plague; 021, Tularemia; 022, Anthrax; 023, Brucellosis; 024, Glanders; 025, Melioidosis; 026, Rat-bite fever; 027, Other bacterial zoonoses; 030, Leprosy; 031, Other mycobacterial disease; 032, Diphtheria; 033, Whooping cough; 034, Streptococcal throat/scarlet fever; 035, Erysipelas; 036, Meningococcal infection; 037, Tetanus; 038, Septicemia; 039, Actinomycotic infections; 040, Other bacterial diseases; 041, Bacterial infection in other diseases not otherwise specified; 090, Congenital syphilis; 091, Early symptomatic syphilis; 092, Early syphilis latent; 093, Cardiovascular syphilis; 094, Neurosyphilis; 095, Other late symptomatic syphilis; 096, Late syphilis latent; 097, Other and unspecified syphilis; 098, Gonococcal infections; 100, Leptospirosis; 101, Vincent’s angina; 102, Yaws; 103, Pinta; 104, Other spirochetal infection; 110, Dermatophytosis; 111, Dermatomycosis not otherwise classified or specified; 112, Candidiasis; 114, Coccidioidomycosis; 115, Histoplasmosis; 116, Blastomycotic infection; 117, Other mycoses; 118, Opportunistic mycoses; 320, Bacterial meningitis; 322, Meningitis, unspecified; 324, Central nervous system abscess; 325, Phlebitis of intracranial sinus; 420, Acute pericarditis; 421, Acute or subacute endocarditis; 451, Thrombophlebitis; 461, Acute sinusitis; 462, Acute pharyngitis; 463, Acute tonsillitis; 464, Acute laryngitis/ tracheitis; 465, Acute upper respiratory infection of multiple sites/not otherwise specified; 481, Pneumococcal pneumonia; 482, Other bacterial pneumonia; 485, Bronchopneumonia with organism not otherwise specified; 486, Pneumonia, organism not otherwise specified; 491.21, Acute exacerbation of obstructive chronic bronchitis; 494, Bronchiectasis; 510, Empyema; 513, Lung/mediastinum abscess; 540, Acute appendicitis; 541, Appendicitis not otherwise specified; 542, Other appendicitis; 562.01, Diverticulitis of small intestine without haemorrhage; 562.03, Diverticulitis of small intestine with haemorrhage; 562.11, Diverticulitis of colon without haemorrhage; 562.13, Diverticulitis of colon with haemorrhage; 566, Anal and rectal abscess; 567, Peritonitis; 569.5, Intestinal abscess; 569.83, Perforation of intestine; 572.0, Abscess of liver; 572.1, Portal pyemia; 575.0, Acute cholecystitis; 590, Kidney infection; 597, Urethritis/ urethral syndrome; 599.0, Urinary tract infection not otherwise specified; 601, Prostatic inflammation; 614, Female pelvic inflammation disease; 615, Uterine inflammatory disease; 616, Other female genital inflammation; 681, Cellulitis, finger/ toe; 682, Other cellulitis or abscess; 683, Acute lymphadenitis; 686, Other local skin infection; 711.0, Pyogenic arthritis; 730, Osteomyelitis; 790.7, Bacteraemia; 996.6, Infection or inflammation of device/graft; 998.5, Postoperative infection; 999.3, Infectious complication of medical care not otherwise classified; 995.91, Sepsis; 995.92 Severe Sepsis;785.52, Septic Shock. Where 3- or 4-digit codes are listed, all associated subcodes were included.

**Procedures ICD-9-CM used in intensive care unit**

Diagnostic procedures on skull, brain and cerebral meninges: 01.10, 01.16, 01.17

Temporary tracheostomy: 31.1

Implantation of heart and circulatory assist system: 37.61, 37.62, 37.68

Insertion of temporary transvenous pacemaker system: 37.78

Arterial catheterization: 38.91

ECMO: 39.65

Dialysis: 39.95, 54.98 with a diagnostic of acute renal insufficiency

Circulatory monitoring: 89.60, 89.62, 89.64, 89.67, 89.68

Respiratory therapy: 93.90, 93.91, 93.99

Non-operative intubation of gastrointestinal and respiratory: 96.04, 96.06

Other irrigation of (naso)-gastric tube: 96.34

Enteral or parenteral infusion of concentrated nutritional substances: 96.6, 99.15

Other continuous invasive mechanical ventilation: 96.70, 96.71, 96.72

Replacement, removal of tracheotomy tube: 97.23, 97.37

Non-operative removal of heart assists system: 97.44

Cardiopulmonary resuscitation, not otherwise specified: 99.60, 99.61, 99.62, 99.63

Aquapheresis: 99.78

Hypothermia (central) (local): 99.81

**ICD-9-CM-based classification of acute organ dysfunction**

Cardiovascular: 785.5, 458.xx

Respiratory: 96.7x

Neurologic: 348.3x, 293.xx, 348.1

Hematologic: 287.4, 287.5, 286.9, 286.6

Hepatic: 570, 573.4

Renal: 584.x

**ICD-9-CM-based classification of site of infection**

*Abdominal*: 001,Cholera; 002, Typhoid and paratyphoid fever; 003, Others Salmonella infections; 003.0, Salmonella gastroenteritis; 004, Shigellosis; 005,Others food poisoning; 008; Intestinal Infections due to others organisms; 009, Ill-defined intestinal Infections; 014 Abdominal tuberculosis; 017.8, Oesophagus Tuberculosis; 022.2 Gastrointestinal Anthrax; 03283, Diphtheritic peritonitis; 101, Vincent ‘s Angina; 540, Acute appendicitis; 541, Appendicitis unqualified; 542, Others appendicitis; 562.01, Diverticulitis of small intestine; 562.03, Diverticulitis of small intestine with haemorrhage; 562.11, Diverticulitis of colon; 562.13, Diverticulitis of colon with haemorrhage; 566 Abscess of anal and rectal regions; 567, Peritonitis and retroperitoneal infections; 569.5, Abscess of intestine; 569.83, Perforation of intestine; 572.0, Abscess of liver; 572.1, Portal Pyemia; 575.0, Acute Cholecystitis.

*Respiratory*: 003.22, Salmonella Pneumonia; 010, Primary tuberculosis Infection; 011, Pulmonary Tuberculosis; 012, Others respiratory tuberculosis; 022.1, Pulmonary Anthrax; 031.0, Pulmonary mycobacteria; 032.0, Faucial Diphtheria; 032.1, Nasopharyngeal Diphtheria; 032.2, Anterior nasal Diphtheria; 032.3, laryngeal Diphtheria; 033, Whooping cough; 034.0,streptococcal sore throat; 039.1, actinomycotic pulmonary Infection; 114.0, Primary Coccidioidomycosi (pulmonary); 114.4, Chronic pulmonary Coccidioidomycosi; 461, Acute sinusitis; 462, Acute Pharyngitis; 463, Acute tonsillitis; 464, Acute Laryngitis and tracheitis; 465, Acute upper respiratory infections of multiple or unspecified sites; 481, Pneumococcal Pneumonia; 482, Others bacterial pneumonia; 485, Bronchopneumonia, organism unspecified; 486; Pneumonia, organism unspecified; 491.21, chronic bronchitis with acute bronchitis; 494, Bronchiectasis; 510, Empyema; 513, Abscess of lung and mediastinum.

*Central Nervous System*: 003.21, Salmonella Meningitis; 013, Tuberculosis of meninges and CNS; 036.0, Meningococcal Meningitis; 036.1, Meningococcal Encephalitis; 090.4, Juvenile Neurosyphilis ; 094, Neurosyphilis; 114.2, Coccidioidal Meningitis ; 320, Bacterial Meningitis; 322, Meningitis of unspecified cause; 324, Intracranial intraspinal abscess; 325, Phlebitis and trombophlebitis of intracranial venous sinuses.

*Genitourinary*: 016, Tuberculosis of genitourinary system; 032.84, Diphtheria Cystitis; 091, Early Syphilis symptomatic; 092, Early Syphilis latent; 095, Others forms of late syphilis , with symptoms; 096, late syphilis latent; 097, Other and unspecified syphilis; 098, Gonococcal Infections; 590; Infections of Kidney; 597, Urethritis not sexually transmitted, and urethral syndrome; 599.0, Urinary tract Infection, site non specified; 601, Inflammatory diseases of prostate; 614, Inflammatory diseases of ovary, Fallopian tube, pelvic cellular tissue and peritoneum; 615, Inflammatory diseases of uterus, except cervix; 616, Inflammatory diseases of cervix, vagina and vulva.

*Endocarditis*: 032.82, Diphtheritic Myocarditis; 036.4 Meningococcal Carditis ; 093, Cardiovascular syphilis; 420, Acute Pericarditis; 421, Acute or sub acute endocarditis .

*Device-related*: 451, Phlebitis and trombophlebitis; 996.6 Infection and inflammatory reaction due to internal prosthetic device, implant and graft; 998.5, postoperative Infection; 999.3 Others infections.

*Wound/soft tissue*: 017.0, Tuberculosis of skin and subcutaneous cellular tissue; 022.0, Cutaneoux Anthrax; 031.1, Cutaneous mycobacteria; 681, Cellulitis and abscess of fingers and toe; 682, Others cellulitis and abscess; 683, Acute Lymphadenitis; 686, Others local infection of skin and subcutaneous tissue.

*Bacteraemia*: 003.1, Salmonella septicaemia; 022.3, Anthrax septicaemia; 027, Others zoonotic bacterial diseases; 031.2, Mycobacteria disseminated; 036.2, Meningococcemia; 036.3, Waterhouse-Friderichsen Syndrome, meningococcal; 038, Septicaemia; 112.5, Candidiasis disseminated; 785.52, Septic Shock; 790.7, Bacteraemia ; 995.91, Sepsis; 995.92 Severe Sepsis.

Others: the rest codes of specific sites from Appendix 1.
